# Women with learning disabilities and Read coding: Lessons from a cohort study

**DOI:** 10.1186/1471-2458-8-252

**Published:** 2008-07-22

**Authors:** Fiona Reynolds, Debbi L Stanistreet

**Affiliations:** 1 Clinical Epidemiology and Public Health Unit, University of Manchester, Manchester, UK; 2 Department of Public Health, University of Liverpool, Liverpool, UK

## Abstract

**Background:**

The aim was to examine any differences in the way that Read codes are applied to the records for female patients with learning disabilities across three PCT areas. To ascertain the most commonly used read codes for learning disability.

**Methods:**

This was a retrospective cohort study carried out in Bury, Heywood-and-Middleton and Rochdale PCTs.

All women in the eligible age-group (25–64) as of the 1^st ^June, 2005, who were in contact with the Learning Disabilities Teams in the relevant PCT areas were identified from the Teams' lists. The appropriate Read Codes were then used to identify women on GP systems. Patient data is stored on the GP database systems (*Vision, EMIS, EMIS PC4 *and *Torex*) and it was possible to search for patients with learning disabilities.

**Results:**

The use of Read Codes varies across the three areas. The most commonly used Read codes were E3 (Mental Retardation) – 27%, PJ0. (Down's Syndrome) – 14% and Eu81z (Learning Disabilities) – 8%. In 24% of the records a Read Code had not been documented.

**Conclusion:**

Read codes application varies between GP surgeries – dependent on PCT policy and the surgery's approach and also as a result of staff time.

## Background

There is conflict over the definitions of the term 'learning disability' – as a result, there is no single definition [1,2]. There is even conflict over whether to use the term *learning disabilities *– the preferred term in the UK [3] and in many parts of the world the phrase *mental retardation *was used for some time [2]. For example, the American Association on Mental Retardation continued to use the term *mental retardation *until as recently 2006 [4]. Oliver [5] points out that the problem of defining *'disability' *is that the word is defined by people who do not have a disability. For example, Crawford *et al*, [6] suggest that definitions should include the phrase *'significant sub-average intellectual functioning' *which represents, in the UK, an IQ below 74. It has taken some time to arrive at 'respectful descriptions' of people with LDs, [7].

The Foundation for People with Learning Disabilities (FPLD) [7] defines learning disabilities as including the presence of significant intellectual impairment; with deficits in social functioning or adaptive behaviour; which are present from childhood.

Confusion often arises over the use of the term *learning disabilities*, because it is often used to refer to conditions that are known as learning difficulties in the UK – such as dyslexia. The Learning Disabilities Forum [8] also suggests dyslexia, attention-deficit hyperactivity disorder and Tourette's syndrome as learning disabilities. However, this is disputed by various other authors [1,2] and although these conditions may be present with learning disabilities [8], on their own are not symptomatic of intellectual impairment, [7]; and do not affect understanding [9].

Research shows that many people with learning disabilities have undetected health conditions that can cause unnecessary pain; or reduce the quality or length of their lives [10].

People with learning disabilities, and their carers, often have low expectations of their own health and the services they receive; often to the extent of tolerating poor health [10]. Women often report that staff lack understanding of their disability and often focus on this rather than the immediate health issue [11]. It can be difficult for people with learning disabilities to access care and treatment, especially screening services [10,12]. Barriers to accessing services include fear of examinations and difficulties in accessing professionals who link to services [13].

In 2006, the Disability Rights Commission (DRC) published its findings on people with learning disabilities and health inequalities. It [14] cited research [15,16] that shows cervical screening uptake rates are much lower in women with learning disabilities – variously estimated at 13% and 47%, as compared with 84 – 89% in the general population. "The reasons for the variation among women with learning disabilities are not known but may be linked to small sample sizes and the less reliable estimates which they produce [14]." As we discussed in our previous paper [17], screening is rarely offered to women with learning disabilities and coverage is lower than for women in the general population [18]. Brent and Harrow Health Authority found that only 19% of women with learning disabilities had received screening while 77% of the women had no screening records, [19]. Pearson *et al*, [20] discovered that 37% of women with learning disabilities were ceased because they had a learning disability. A recent study carried out by Smith [21] in Rochdale suggests that General Practitioners (GPs) considered cervical screening unnecessary for women with learning disabilities.

The DRC [14] recommended that screening programmes are targeted in line with evidence and are fully inclusive of people with learning disabilities, and that improvements should be made through the commissioning process. A number of studies found that women with learning disabilities may not come for screening for various reasons and recommended that primary care staff adhere to guidelines and work with the women to encourage them to use the services [22,23].

The Department of Health publication, *Valuing People *[10] sets out how the NHS may contribute to reducing the health inequities suffered by people with learning disabilities through improving access to services. It stated that:*"By June 2004, all patients with learning disabilities should have been coded onto primary care data systems." *The issue of services for patients with learning disabilities is now also a Quality and Outcomes Framework target for all surgeries. Since 2006, practices have had to be able to produce a register of patients with learning disabilities.

There are thousands of Read codes and as new developments, diagnoses, techniques and suggestions by users come into practice, new codes are released bi-annually to allow updating [20]. Approximately 87% of medical practices are computerised, and of these 80% use Read codes – which is expected to rise to above 90% in the next three years.

Among the advantages of the Read codes are that they are simple to implement and can aid searches. They also allow us to record data more consistently; retrieve it more easily and analyse more thoroughly.

## Methods

A retrospective cohort study, using case control methods, was carried out to compare the uptake of cervical screening and the likelihood of being ceased, between women with and without learning disabilities. The use of Read coding was also explored as a part of this work to ascertain the most commonly used codes for learning disability. The study population included women aged 25–64 with learning disabilities in the PCT areas of Bury, Heywood and Middleton and Rochdale.

Using *Epi.Info Stat.Calc*, we calculated that a sample size of 217 women with learning disabilities and 434 women without was required assuming prevalence of screening to be 80% and based on an OR of 2, and a sample ratio of 1:2 (women with learning disabilities to women without) Significance was set at 0.05 and power at 80%. Looking at Read coding for learning disabilities, we focused on the case group and we found that there were actually 267 women with learning disabilities who were in contact with the Learning Disabilities Teams or the GPs in the three areas.

### Design and Process

This study was undertaken across Bury, Heywood-and-Middleton and Rochdale PCTs. There were 34 GP practices in Bury, 21 in Rochdale and 14 in Heywood-and-Middleton and work with these took place September-December, 2005. Patient data were stored on the database systems *Vision, EMIS, EMIS PC4 *and *Torex*.

The following Read Codes (used for diagnoses and treatment) were used to identify women on GP systems identified as having learning disabilities. The reason for carrying out this second process of ascertainment was to ensure that as complete a list as possible, of women with learning disabilities, was obtained.

The named learning disabilities are listed in Table 1.

**Table 1:** ICD10 codes for learning disabilities mapped to Read Codes and their definitions.

**Read Codes**	**Definitions**
Eu81z	Learning Disability
E3	Mental Retardation
E30	Mild Mental Retardation
E310	Moderate Mental Retardation
E311	Severe Mental Retardation
E312	Profound Mental Retardation
E140	Autism
Eu842	Rett's Syndrome
N726./PKy93	Prader-Willi Syndrome
N721/PJ0../PJ0z	Down 's syndrome
N724/PJ2../PJ2z	Edward's Syndrome
PKyz5	Angelman Syndrome
N725/PJyy4	Fragile × Syndrome
C301	Phenylketonuria

**Ethics Committee: **North Manchester LREC 05/Q1406/82

### Obtaining Consent for Records to be Accessed

This study was carried out by accessing patient records. Although people caring for a woman with learning disabilities (carers), whether family or a paid employee, cannot consent on her behalf, the Mental Capacity Act (2005) advises that they (or nominated third parties) should be consulted to discover whether the person with learning disabilities would assent to joining any research project. A letter was sent to 267 women with learning disabilities requesting their permission to access their records. In the event that the women did not understand the letter, it was anticipated that they would pass it to their carer. In some cases, carers/parents contacted the PCT office to enquire further about the study.

The letter stated that if the women (or their carers) did not wish to give permission they should contact the study. 46 people contacted the office. The carers of four women withheld consent on the grounds that the woman could not consent. Of the 42 people who wished to find out more 37 were parents who stated that their daughters had never had a screening test but that the women assented to being included in the study for checking. Five were women with learning disabilities who wished to know more and gave their consent.

## Results

The use of Read Codes varies across the three areas. The most commonly used Read codes were E3 (Mental Retardation) – 27%, PJ0. (Down's Syndrome) – 14% and Eu81z (Learning Disabilities) – 8%.

In Bury and Heywood-and-Middleton the largest code proportion was actually 'unknown' i.e. the Surgeries had not yet Read coded the learning disabilities of these women. In Rochdale, this was the second largest share. Rochdale and Heywood-and-Middleton PCTs had written to all GPs to instruct them to code all patients with learning disabilities under the codes E3 (mental retardation) and Eu81z (learning disabilities). These do not tell us whether there are any particular learning disabilities (e.g. Down 's syndrome) which may be common or unusual (Table 2).

**Table 2:** The numbers and percentages of women with learning disabilities with each Read code.

**Read Code**	**Definition**	**Number of women (n = 290)**
**Unknown**	Code has not been given	70 (24%)
**Eu81z**	Learning Disabilities	23 (8%)
**E3**	Mental Retardation	79 (27%)
**E30**	Mild Mental Retardation	4 (1%)
**E311**	Severe Mental Retardation	8 (3%)
**PJ0/PJ0z**	Down's Syndrome	41 (14%)
**C301**	Phenylketonuria	16 (6%)
**E140**	Autism	1 (0.005%)
**Eu842**	Rett's Syndrome	1 (0.005%)
**E3 + Another Code**	Two codes have been given with E3 as the main code	16 (6%)
**Eu81z + Another Code**	Two codes have been given with Eu81z as the main code	31 (11%)

After each visit to a Surgery where the women with learning disabilities had not received a Read code, the LD Teams were notified so that they assist in the process.

E3 was the most commonly used code – probably as a result of coding policy in Rochdale and Heywood-and-Middleton, which makes it surprising that Eu81z took such a small share.

Down 's Syndrome was shown to be the next most commonly coded condition – especially in Bury, but not in the other two areas. However, it is likely that this is an artefact as a result of policy rather than a genuine increase.

## Discussion

### "How this fits in"

The Department of Health publication *Valuing People *(2001) sets out how the NHS may improve access to health services for people with learning disabilities – specifically women's access to screening and examines how accurately their needs have been coded. A specific recommendation was that by June 2004, all patients with learning disabilities should have been Read coded onto primary care data systems. This study explored whether this target had been reached. The QOF target of every practice producing a register of patients with learning disabilities was introduced in 2006 – this study was carried out before this came into force.

### Main Findings

It is likely that the findings are comparable to the experiences of other PCTs.

The blanket use of generic codes means that it impossible to use GP records to easily identify any common types of learning disability or clusters. This could impede the provision of any specific specialist support that could be offered to patients, and the improvement of services, because the generic codes mask the situation. However, it should be noted that large 'clusters' were found at a number of practices, usually where there was a care facility nearby.

The decision to encourage GPs to code patients with learning disabilities solely under these two Read codes is interesting. It is likely that this was done to ease the workload of staff responsible for Read-coding patients with learning disabilities; as *Valuing People *[14] imposed a deadline of June 2004 for coding patients.

### Strengths and Limitations of the Study

A key weakness of this study is that it focused only on women – learning disabilities are more common among men. It is also possible that the selection of Read codes used may have distorted one aspect of the Study's findings and weakened the work. The Read Codes used in this Study were selected on advice from the Learning Disabilities Teams (LD Teams) and other PCT staff. It was not an exhaustive list. Other codes were omitted to ensure that women without learning disabilities were not considered among the figures (e.g. cerebral palsy is often included in lists). The use of Read codes can be problematic as one description can have two, even three codes. This *could *confound any audits or research as the codes inputted to GP records will vary according to which operator carries this work out.

The actual number, of women whose Read code for learning disabilities is 'unknown', *may *be smaller than that reported here. Because a limited number of codes were searched for, it is possible that the women who were 'not coded' were coded under another definition.

Having carried out the research in discussion with the practice managers it is likely that this has not greatly distorted the data. Many of the managers reported that there had not been the time or staff to Read-code patients appropriately. Furthermore, previous instruction from Heywood-and Middleton and Rochdale PCTs, to code women with learning disabilities under the codes of E3 (Mental Retardation) and Eu81z (Learning Disabilities), should have reduced the numbers of women identified in the two areas as being 'not coded'. The lack of completeness however does show that more work needs to be done in certain practices to either update their records or improve access to screening for women with learning disabilities.

While it would have been simpler if all records had been available from one central point, it was useful to visit the practices as discussion with practice managers provided more information about identifying women who had not been Read coded.

Identifying women with learning disabilities by Read codes meant that records could be identified without having to trawl through thousands of others. Searching by codes meant that only the relevant aspects of the patients' records were viewed. All other personal details were hidden, thus confidentiality was not broken.

### Comparison with Existing Literature

The [1] assertion that GPs have no idea of the numbers on their lists was shown to be incorrect, here – certainly by practice managers, who knew their patient lists very well. However, this assertion was made prior to the new GP contract and GPs now have a very good idea, in many cases very accurate idea of how many of their patients have learning disabilities.

### Implications

The lack of Read coding is an issue that was addressed as a result of this study. Once a practice was visited the LD Teams were notified if there were gaps in coding so that they could assist. The lack of coding shows that the practices have not been able to meet the deadline of coding by 2004, which may have a 'knock-on' effect on further work dependent on this being completed.

## Conclusion

Read codes application varies between GP surgeries – dependent on PCT policy and the surgery's approach and also as a result of staff time.

Although practices know who their patients are and understand their needs, this information had not been coded into the records which would make retrieval and provision of support easier in the long term.

It may be more useful for surveillance (as well as future audits and research) if GP surgeries coded patients with learning disabilities with specific codes. Being able to identify learning disabilities, both as specific conditions (e.g. Down's Syndrome) and as a gauge of the severity of impairment (e.g. scale of mild to profound learning disabilities), would support tailoring of services specific to patient needs, as well as potentially identifying unusual clusters.

*Valuing People *[10] states that it is necessary for patients with learning disabilities to have personal health plans, and LD Teams could provide support in developing and updating these.

## Competing interests

The authors declare that they have no competing interests.

## Authors' contributions

FR carried out the study and wrote this report. DLS supervised the study, providing support and guidance.

## Pre-publication history

The pre-publication history for this paper can be accessed here:


